# Supplementary material for: CNEr: A toolkit for exploring extreme noncoding conservation
Source: PLoS Comput Biol. 2019 Aug 26;15(8):e1006940. doi: 10.1371/journal.pcbi.1006940 (PMC6730951; doi:10.1371/journal.pcbi.1006940)
Supplement: S1 Text — (PDF) [file pcbi.1006940.s001.pdf]

S1 Text: A comparison of CNEs identified by PHAST and CNEr on human

To compare the performance of PHAST and *CNEr*, we generated hg38 CNEs from these two methods. We downloaded hg38 multiz7way alignment from UCSC and ran phastCons with following parameters: expected-length=50, target-coverage=0.125, rho=0.3. Please note that the parameters used here are different from the UCSC version: expected-length=45, target-coverage=0.3, rho=0.3, in order to keep the most conserved elements. In the end, PHAST produced 750,714 conserved elements. Aftering filtering out the coding/repeats region and elements shorter than 50bp, 343,728 elements were retained. In contrast, 65,483 CNEs were identified from hg38-mm10 alignments with 98% over 50bp criteria by *CNEr*. The venn diagram of these two sets of CNEs are shown below, with "within" (The cross area is the number of CNEr CNEs are completely within PHAST CNEs) and "overlap more than 50bp" (The cross area is the number of *CNEr* CNEs that overlap at least 50bp with PHAST CNEs). As we can see, the majority of *CNEr* CNEs are shared with PHAST. The very few *CNEr* specific CNEs are due to varying reasons. For instance, the CNE can be lost or missing from the genome assembly in species other than mouse. Some conserved elements are not called from 7way alignments, although they are considered as conserved elements in 20way alignments.

In short, the PHAST scoring model can identify elements at lower level of conservation, but their score will not bear a simple relationship to that of the conservation level of CNEs from pairwise alignments. Instead, elements with lower conservation across multiple species in the alignment will score higher than elements with high sequence similarity across only a subset of species. This makes it difficult to set a meaningful score threshold for subsequent analysis such as determination of CNE cluster boundaries or for studying CNE turnover.

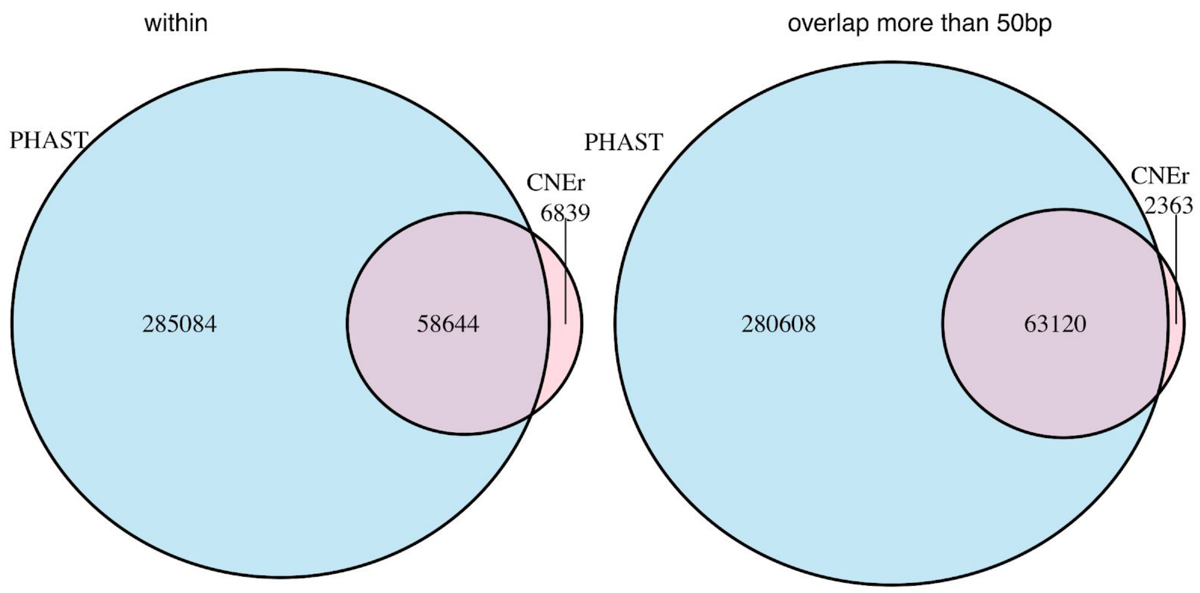
within
PHAST
CNEr
285084
58644
6839
overlap more than 50bp
PHAST
CNEr
280608
63120
2363
