# Supplementary material for: CNEr: A toolkit for exploring extreme noncoding conservation
Source: PLoS Comput Biol. 2019 Aug 26;15(8):e1006940. doi: 10.1371/journal.pcbi.1006940 (PMC6730951; doi:10.1371/journal.pcbi.1006940)
Supplement: S2 Text — (PDF) [file pcbi.1006940.s002.pdf]

S2 Text: Working with Paired Genomic Ranges

In Bioconductor, the *GRanges* class defined in the *GenomicRanges* package [1] is an essential class that encodes the "start" and "end" position of ranges, as well as the chromosome identifier, strand designation and other metadata. Due to the nature of conducting pairwise comparison between species, we needed a class that stores two parallel *GRanges* classes, which represent the genomic coordinates information from each species. With direct inheritance from *Pairs* class of *S4Vectors* package, we created a *GRangePairs* class. The only restriction for these two *GRanges* objects is that they must have same lengths. They can represent data from the same genome or different genomes. Since the CNE identification algorithm scans the conserved elements from axt alignment files, it is important to be able to manipulate the axt alignment efficiently in R. We build another Axt class on top of GRangePairs with shared element metadata, including Blastz score, alignment length and alignment sequence, for each pair. This *Axt* class can be especially useful for comparative genomics and phylogenetic footprinting [2]. With the inheritance from *Pairs*, many common Bioconductor Vector APIs are preserved for convenient operations. More details about the specific methods defined for *GRangePairs* and *Axt* class are available in the documentation or vignette that comes with our package.

References:

1. Lawrence M, Huber W, Pagès H, Aboyoun P, Carlson M, Gentleman R, et al. Software for computing and annotating genomic ranges. PLoS computational biology. 2013. p. e1003118.
2. Tan G, Lenhard B. TFBSTools: an R/bioconductor package for transcription factor binding site analysis. Bioinformatics (Oxford, England). 2016;32: 1555–1556.
