# Supplementary material for: CNEr: A toolkit for exploring extreme noncoding conservation
Source: PLoS Comput Biol. 2019 Aug 26;15(8):e1006940. doi: 10.1371/journal.pcbi.1006940 (PMC6730951; doi:10.1371/journal.pcbi.1006940)
Supplement: S3 Text — (PDF) [file pcbi.1006940.s003.pdf]

S3 Text: Glossina and sea urchin data

The *Glossina morsitans* genome assembly was obtained from Sanger Institute release December 2010 and the gene set version GmorY1.5 was acquired from VectorBase (https://www.vectorbase.org) [1]. This 366-Megabase *Glossina* assembly contains 13,807 scaffolds with a N50 value of 120 kb. This genome size is more than twice the size of *D. melanogaster* genome. 12,308 protein-coding genes were predicted and the average gene size is almost double of that of *Drosophila*. The average exon and intron sizes are 491 bp and 1.6 kb, respectively. The whole genome pairwise alignment between *Drosophila* and *Glossina* is generated by our *LASTZ* pipeline with the parameter of distance="far".

The sea urchin *Strongylocentrotus purpuratus* v3.1 and *Lytechinus variegatus* v2.2 genome assemblies and gene annotations were downloaded from *EchinoBase* (http://www.echinobase.org/Echinobase/) [2]. The number of scaffolds are 32,008 and 322,794, respectively. Due to the highly fragmented assemblies, the whole genome pairwise alignment was done with *LAST* pipeline with parameter of distance="far".

References:

1. International Glossina Genome Initiative. Genome sequence of the tsetse fly (Glossina morsitans): vector of African trypanosomiasis. Science. 2014;344: 380–386.
2. Cameron RA, Samanta M, Yuan A, He D, Davidson E. SpBase: the sea urchin genome database and web site. Nucleic acids research. 2009;37: D750–D754.
