# Supplementary material for: CNEr: A toolkit for exploring extreme noncoding conservation
Source: PLoS Comput Biol. 2019 Aug 26;15(8):e1006940. doi: 10.1371/journal.pcbi.1006940 (PMC6730951; doi:10.1371/journal.pcbi.1006940)
Supplement: S4 Text — (PDF) [file pcbi.1006940.s004.pdf]

S4 Text: GRB boundaries identification

A GRB is defined as a cluster of syntenic CNEs around a key developmental target gene. In this project, we use a automated CNE clustering pipeline for estimating the GRB boundaries. Given CNE locations as the only input, so the algorithm tries to identify the spans of high density of CNEs above the expected CNE density, effectively segmenting the genome into GRBs and regions outside GRBs. The CNE density is calculated with a smoothing window of 40kb for *Drosophila* and sea urchin. The GRB is also required to have more than 10 CNEs. The implementation is available as "makeGRBs" in *CNEr* package.
