# Supplementary material for: CNEr: A toolkit for exploring extreme noncoding conservation
Source: PLoS Comput Biol. 2019 Aug 26;15(8):e1006940. doi: 10.1371/journal.pcbi.1006940 (PMC6730951; doi:10.1371/journal.pcbi.1006940)
Supplement: S1 Algorithm — (PDF) [file pcbi.1006940.s012.pdf]

Algorithm S1. The algorithm of scanning axt alignment.

```
algorithm ceScan(axt, filtersTarget, filtersQuery, I, W)
  input:
    axt: Axt alignment
    filtersTarget: ranges to filter of target assembly
    filtersQuery: ranges to filter of query assembly
    W: the window size of the running window.
    I: the minimal identity over the winSize
  output: ranges of CNEs
HT <- new Hashtable from filtersTarget
HQ <- new Hashtable from filtersQuery
for each alignment a in axt:
  initialise temp alignment t
  for each running window w of size W in a:
    if HT contains w or HQ contains w:
      goto next w
    end
    if identity of w > identity:
      append w to t
    end
  end
  merge overlapping w in t
  return t
end
```

Algorithm S1: Scan axt alignments and identify the conserved noncoding elements

---
