# Supplementary material for: CNEr: A toolkit for exploring extreme noncoding conservation
Source: PLoS Comput Biol. 2019 Aug 26;15(8):e1006940. doi: 10.1371/journal.pcbi.1006940 (PMC6730951; doi:10.1371/journal.pcbi.1006940)
Supplement: S1 Code — (ZIP) [file pcbi.1006940.s013.zip › S1Code/package/inst/doc/CNEr.html]

CNE identification and visualisation


# CNE identification and visualisation

Ge Tan

#### *31 May 2018*

#### Abstract

Comparative genomics has revealed noncoding DNA regions with extremely high conservation across large evolutionary distances. These regions have been termed conserved noncoding elements (CNEs). Despite of various resources of CNEs, there is no publicly available tool to identify these elements from scratch. We describe CNEr, an R package for large-scale identification and advanced visualisation of sets of CNEs from *Axt* alignments. Given whole genome pairwise alignments of two species as input, our pipeline facilitates alignment screening, elements merging, calculation of CNE density and visualisation of CNEs in the form of horizon plots. Furthermore, it provides efficient scalable data structures for representing paired ranges on the genome, especially the support for pair alignments in addition to functions potentially useful for exploratory analysis of the identified elements.

#### Package

CNEr 1.16.1

# Contents

- 1 Introduction
- 2 Workflow of the package
  - 2.1 CNE identification
  - 2.2 CNE visualisation
- 3 Input
  - 3.1 axt alignment file
  - 3.2 Filtering information
  - 3.3 Creating a `CNE` class
- 4 CNE identification
  - 4.1 Scan axt alignments
  - 4.2 Merge CNEs
  - 4.3 Realignment of CNEs
  - 4.4 CNE storage and query
  - 4.5 CNE length distribution
  - 4.6 Genomic distribution of CNEs along the chromosome
  - 4.7 Output of bed and bedGraph files
- 5 CNEs visualisation
  - 5.1 Gene annotation visualisation
  - 5.2 CNEs horizon plot
- 6 Conclusions
- References

# 1 Introduction

Conserved noncoding elements (CNEs) are a pervasive class of elements
clustering around genes with roles in development and differentiation
in Metazoa (Woolfe et al. 2004).
While many have been shown to act as long-range developmental enhancers
(Sandelin et al. 2004),
the source of their extreme conservation remains unexplained.
To study the evolutionary dynamics of these elements
and their relationship to the genes around which they cluster,
it is essential to be able to produce genome-wide sets of these elements
for a large number of species comparisons,
each with multiple size and conservation thresholds.

This *CNEr* package aims to detect CNEs and
visualise them along the genome.
For performance reasons, the implementation of CNEs detection
and corresponding I/O functions are primarily written as C extensions to R.
We have used *CNEr* to produce sets of CNEs by scanning pairwise whole-genome net alignments
with multiple reference species,
each with two different window sizes and a range of minimum identity thresholds.
Then, to pinpoint the boundaries of CNE arrays,
we compute the CNE densities as the percentages of length
covered by CNEs within a user specified window size.
Finally, we describe a novel visualisation method using horizon plot tracks
that shows a superior dynamic range to the standard density plots,
simultaneously revealing CNE clusters characterized
by vastly different levels of sequence conservation.
Such CNE density plots generated using precise locations of CNEs
can be used to identify genes involved in developmental regulation,
even novel genes that are not annotated yet.

# 2 Workflow of the package

This section briefly demonstrates the pipeline of CNE identification
and visualisation.
More detailed usage of each step is described in following sections with a concise example of CNE identification and visualisation for the “barhl2” and “sox14” (chr6:24,000,000..27,000,000) loci in Zebrafish (danRer10) genome against Human (hg38).

## 2.1 CNE identification

1. Preparation of axtNets: the axtNet files can be downloaded from UCSC
   or generated by this package.
2. Identification of conserved noncoding regions: scan the axt alignments for the regions, with minimal **I** identities over **C** columns, which do not overlap with annotated exons/repeats.
3. Merging overlapped elements : scanned elements from two pairs of alignments (one from each genome as reference) that overlap on both genomes are merged.
4. Removal of unannotated repetitive sequences: realign the elements back to respective genome with *BLAT* and discard the elements which exceed a certain number of hits on either genome.

## 2.2 CNE visualisation

1. Display parameters: chromosome, start, end, smooth window size.
2. Horizon plot: visualise CNE densities with the package *Gviz*.

# 3 Input

The minimal input for *CNEr* includes the whole genome pairwise alignment of two assemblies, axt
net files.
UCSC already provides a set of precomputed axt files on http://hgdownload.soe.ucsc.edu/downloads.html for most of popular genomes.
In case the *axt* net files are not available from UCSC, you can always generate the axt net files by following another vignette “Pairwise whole genome alignment” in this *CNEr* package.

Another essential information is the annotation of exons and repeats, which could be retrieved from various resources.

## 3.1 axt alignment file

During the development of this package, there was no suitable class to store the axt alignments in Bioconductor.
Hence, we created two new **S4** classes, `Axt` and `GRangePairs`, to easily manipulate the axt alignment files.

`GRangePairs` is designed to hold a pair of `GRanges` objects, which have the same length.
It builds on the `Pairs` class of Bioconductor and inherits many useful methods from it.
This `Axt` class inherits from `GRangePairs` with no extra slots to hold the content from *axt* files, but many specific methods are created for `Axt`.
The ranges of the target and the query organism are stored in two `GRanges` objects with alignment sequences as metadata columns.
The Blastz scores and the widths of the alignments are stored in metadata columns of `GRangePairs`.
For more information on the usage of these two classes, please refer to the documentation.

To read axt file into R,
*CNEr* provides `readAxt` function for highly efficient reading.
This function is built on a backend C code of Kent’s utilities (W J Kent et al. 2002).
The axt alignment files can be either gzippped or in plain text file.
The alignments between two genomes can also be in one big file or in several files, such as “chr1.hg19.mm10.net.axt.gz”, “chr2.hg19.mm10.net.axt.gz”, etc.

```
library(CNEr)
## These axt files are specially prepared for the region
## (chr6:24,000,000..27,000,000)
axtFilesHg38DanRer10 <- file.path(system.file("extdata", package="CNEr"), 
                                  "hg38.danRer10.net.axt")
axtFilesDanRer10Hg38 <- file.path(system.file("extdata", package="CNEr"), 
                                  "danRer10.hg38.net.axt")
```

```
axtHg38DanRer10 <- readAxt(axtFilesHg38DanRer10)
```

```
## The number of axt files 1
## The number of axt alignments is 50
```

```
axtDanRer10Hg38 <- readAxt(axtFilesDanRer10Hg38)
```

```
## The number of axt files 1
## The number of axt alignments is 352
```

```
## Axt class is shown in UCSC axt format
axtHg38DanRer10
```

```
##  A Axt with 50 alignment pairs:
##  1  chr1 148165963 148166304 chr6 25825774 25826099 +  5310 
## TCTCTCCCATCCCAACTGTTCTAAATGT-TCTTCCAT...CAGCTCTTTTCCAACCAAAACAACAGAAATTAAACTT
## TTTCTCTCCTGCTAGGTTTTATAAGAGTGTTTTTCAG...CGGGTGCCTTTTCAGAGTAATGAGGGAAATTAAATTT
##  2  chr1 222131480 222131835 chr6 24819722 24820074 +   221 
## CTTAATGTCTTTCTATCCAAAGTCATGCATTATATGT...AATTGTGTTTAATATTTTTAATTAAATGGTCCTTTTA
## TTCGAATTTTTATTATTAAGATTCAAATATTATG-AT...TACTGTATGTAACAGCATTTATTAAACTGTGTTATCA
##  3 chr10  65322021  65322919 chr6 26227619 26228468 +   946 
## TTGCACTTTGAAGCCCAAACTTAGCAATAAATAAAAA...CTGTTACATTGAGCCATTTGTCTCACTTTTCATTAAT
## TTGTTTTTAAAAGACTAAATTTAACAATAAACTTAAA...TTTGAAAAGTAATTTTTTTGTATTTATATTCATTCAT
##  4 chr13  94750629  94751259 chr6 26600600 26601208 +  3302 
## GCCTTCCCAGTTTCTCC-ACTCTCCCTCATTTTTTTC...TCATAATATA---CATTTTTAAAATTAATTATCCATT
## GCTTTATCAGACTCTCCTAATGTCACCC---TCTCTC...CCAAAAGATAGGGCATTCTAAAACTGTGTCACCCACT
##  5 chr13  94966940  94966991 chr6 26745445 26745499 +   711 
## ATTCAGCTCTAAAGCACCTTTGACTTCTA---TTTCATTTTTTATTTTCCACTCA
## AGCCAGCGTGTATACCTAGCTGACATTTAGGTTTTTTTTTTTTTTTTGCCATTTA
## ...   ...       ...       ...  ...      ...      ...   ...
## 46  chr6  61961895  61962455 chr6 24832605 24833189 + -3626 
## AAACGGGATCATTCTGGCAG---CTGTGTTTAAAA--...TTCAGTAGTTCCCAATCTATGGGCTGCACACTCTAAG
## AAAGGAAATAATTGTTGAAAGTACTGAATTTAAAGAA...TTTATCTGTTTTGAACAAAT----TTAACA----AAA
## 47  chr6 131822466 131822664 chr6 25067163 25067383 +  1420 
## AAAAATACTATCTATTGCAAA-----AAATTCAGAAA...CTTT-----TTTCATTTATATTCCATTATGAAAGTTT
## AAATAGGCTATATATGTCATACTTTTAAACTTTTACA...CTTTTTTTCTTTCATTTATGACATATTATAGTATTTT
## 48  chr7  16091079  16091261 chr6 24823861 24824043 +  1243 
## TACAATTTAAAAACAGAAA-AGAAGGAAATGACATCT...ACACAAGACAAAAAGACAAAATATCACCTGGTGACAG
## TGCAATTTAAAAGTATAAATAAACAGACAATTTATCT...AATAATGATATATAAACAAAATGTGACCTAATAACAG
## 49  chr8 111351278 111351934 chr6 25339132 25339742 +  3524 
## TATAGCAGTGTGAAAATGAACTAATACACATACAA--...GAAATAAAGATAGTATTTGAAGTTTAAACAGAGGAAG
## TATTATATTTGGGAACAGAACCAACAAAATTATGATG...TTAATTAAAATAATATTTAAAATGAACAGATAGAAAG
## 50  chr8 111354557 111354869 chr6 25340098 25340413 +   722 
## TTCTGATAAAC---TTCAGGGTTCCCATCTCAAGATC...AAGGACAAATAAATAGGTGCTGAAGAGAAAAGCTAGA
## TGCTGATTAGCGCTCTCACGTTGCCTGCCATGAGATT...AAGAAAAAATAAAGAGTGTCTGAAAGGAAAAACTAAA
```

```
axtDanRer10Hg38
```

```
##  A Axt with 352 alignment pairs:
##   1 chr6 24000620 24001357 chr7 146767015 146767753 - -4315 
## GCGGATTTATGTAGAAAGATTTTTGGGAGTATCATAA...GAGCCACAATTCTCATTCAGCTATGTGTAGGGTTATT
## GAGGGCCAGTCTGGAAAGCTAACTAGGAGGATGCCAA...AGAAAGGGATTCATAATGGAAACTGAGGGAGCTGCTT
##   2 chr6 24001358 24001524 chr9  17091651  17091817 -  8118 
## ACCTTGTATTTAATTCCAGTTTTAAAATAGCCAAGGA...CACTTTCATCCTGAGAACACTCATTACCTGCAGTTCA
## ACCTTGTACTTCAGGCCAGACTTGAAGTAGCCTAGGA...CGCTCTCATCCTGGCTGCACTCATTGCCTACGGGACA
##   3 chr6 24001525 24001683 chr7 146767937 146768119 -  -142 
## AACA------GACACAGAGTATTG----CCAATATCT...--ACAGTACTTCTGTCAGTCTTATTTCTTTTATTTTG
## ATTATCAAGCAAGAGAAAGGATTAAGGCAGAATTTCT...AATTAACAATTTTTTTAGTCTATTTTTTTTTTTTTTG
##   4 chr6 24001998 24002188 chr9  17092546  17092748 -  5826 
## CTCACCCAGCCACATATGGACATTATAGGACGGAG--...CCTGCATTCTGGAACTCCTTGTGAAAAACCATGATTC
## CTCACCCAGCCAGTAGTGGAGGTCATACTGCAGATTT...CCTGCCTTGAGGAACTCGGGGTGTTCCACCACCATGC
##   5 chr6 24052977 24053618 chr1 156772050 156772641 - 15004 
## AACAAAAGGAAGAAAGGGATAACCCTGTCCAG---TT...TCCTTATGTGGGCTTAAAAAAAAAAAACACACTGGAA
## AAAAAATAAAGGAGTGGCATAAATTTTTCCAAAATGT...TACCCTCAGAGTTTTGTAGAATATTAACACAATATAA
## ...  ...      ...      ...  ...       ...       ...   ...
## 348 chr6 26901575 26901753 chr2 241264446 241264624 +  5495 
## ACCTGGGTGATGATTGAGGACTTCAGAGGTTTAATCT...CACTGCTAGGGTCTCCTCCTGCAGCAGCTAATGAAGA
## ACCTGAGTGATGACAGAAGCCTTGATGGGTCGGATCT...AATT--TAGAGTGGCA-ACTGGACCAGCCAACACAGA
## 349 chr6 26901818 26902021 chr2 241266790 241266977 +  4753 
## TCACCTTTGACCTGCTCTGGCAGTAAGCCACTGCGAT...CAACGTTCGGCATATTTGTAAAGTTACAACCATAAAA
## TCACCTTTGATTTGTTGCGGAACCAGCCCACTTCGGT...CAACCCTCAAT-TATCTATGAGATTGTTAACCTAAGA
## 350 chr6 26903886 26904002 chr2 241268465 241268573 +  4643 
## GGTCAATCTTACCAGCTAAATGGTCAGTTACCAGATA...TTGCCTAAAAACAAAACAGTAAATAAGAGAAGAAAAA
## GGTCAAACTTACCAGCAAAACGGTCAGTAGCCAGAAG...TTGCT--GGTATGGGATGGGAAGTGGGAGAGGAGAGA
## 351 chr6 26996747 26997678 chr6 108023708 108024516 -  1325 
## ATTGTAAATAATACACTGTGCATTACAAATGCATATC...ATTTCTTGTAGTCAAAAGCGGATGGGCAACTTCTTCT
## ATTATATATTATATATTGTACTACATATACATATATT...ATTTCTTG----CAATATCATATGCAAAATTCCTTAT
## 352 chr6 26999619 26999997 chr6 108026981 108027383 -   579 
## TTTTTAACTGACAAGTGACACCAAGAATATTAAT---...TGCTGGCCATCTGTTTTTGAGTTTGCCAATCCAGCTT
## TTTCTGAGTCGCAAATAAAAAATAAAATATAAATGGG...AGTTAACTATCTTTGGTTGGCTTAATGAATCCAGTTT
```

```
## Distribution of matched alignments; Given an Axt alignment, plot a heatmap with percentage of each matched alignment
matchDistribution(axtHg38DanRer10)
```

```
matchDistribution(axtDanRer10Hg38)
```

```
## Example of chr4 on hg19 and galGal3
## The synteny of human and zebrafish is not quite obvious on the dotplot.
library(BSgenome.Hsapiens.UCSC.hg19)
library(BSgenome.Ggallus.UCSC.galGal3)
fn <- file.path(system.file("extdata", package="CNEr"),
                "chr4.hg19.galGal3.net.axt.gz")
axt <- readAxt(fn, 
               tAssemblyFn=file.path(system.file("extdata",
                                     package="BSgenome.Hsapiens.UCSC.hg19"),
                                     "single_sequences.2bit"),
               qAssemblyFn=file.path(system.file("extdata",
                                     package="BSgenome.Ggallus.UCSC.galGal3"),
                                     "single_sequences.2bit"))
```

```
## The number of axt files 1
## The number of axt alignments is 14411
```

```
library(GenomeInfoDb)
syntenicDotplot(axt, firstChrs=c("chr4"), secondChrs="chr4", type="dot")
```

There are methods defined for handling `Axt` objects, including subsetting, output to axt files.
More details can be found in the man page.

## 3.2 Filtering information

The gene annotation information, including exons and repeats, is used to filter out the undesired regions.
Here we summarise a table of filtering information we used:

| Assembly | Name | Exon | Repeat |
| --- | --- | --- | --- |
| hg38 | Human | RefSeq Genes, Ensembl Genes, UCSC Known Genes | RepeatMasker |
| mm10 | Mouse | RefSeq Genes, Ensembl Genes, UCSC Known Genes | RepeatMasker |
| xenTro3 | Frog | RefSeq Genes, Ensembl Genes | RepeatMasker |
| tetNig2 | Tetraodon | Ensembl Genes |  |
| canFam3 | Dog | RefSeq Genes, Ensembl Genes | RepeatMasker |
| galGal4 | Chicken | RefSeq Genes, Ensembl Genes | RepeatMasker |
| danRer10 | Zebrafish | RefSeq Genes, Ensembl Genes | RepeatMasker |
| fr3 | Fugu | RefSeq Genes | RepeatMasker |
| anoCar2 | Lizard | Ensembl Genes | RepeatMasker |
| equCab2 | Horse | RefSeq Genes, Ensembl Genes | RepeatMasker |
| oryLat2 | Medaka | RefSeq Genes, Ensembl Genes | RepeatMasker |
| monDom5 | Opossum | RefSeq Genes, Ensembl Genes | RepeatMasker |
| gasAcu1 | Stickleback | RefSeq Genes, Ensembl Genes | RepeatMasker |
| rn5 | Rat | RefSeq Genes, Ensembl Genes | RepeatMasker |
| dm3 | D. melanogaster | RefSeq Genes, Ensembl Genes | RepeatMasker |
| droAna2 | D. ananassae |  | RepeatMasker |
| dp3 | D. pseudoobscura |  | RepeatMasker |
| ce4 | C. elegans | RefSeq Genes | RepeatMasker |
| cb3 | C. briggsae |  | RepeatMasker |
| caeRem2 | C. remanei |  | RepeatMasker |
| caePb1 | C. brenneri |  | RepeatMasker |

For the sake of simplicity, all the information listed above can be fetched easily
with Bioconductor package *rtracklayer*, *biomaRt* and
precompiled Bioconductor annotation packages.
A few examples are given here:

```
## To fetch rmsk table from UCSC
library(rtracklayer)
mySession <- browserSession("UCSC")
genome(mySession) <- "hg38"
hg38.rmsk <- getTable(ucscTableQuery(mySession, track="RepeatMasker",
                                     table="rmsk"))
hg38.rmskGRanges <- GRanges(seqnames=hg38.rmsk$genoName,
                            ## The UCSC coordinate is 0-based.
                            ranges=IRanges(start=hg38.rmsk$genoStart+1,
                                           end=hg38.rmsk$genoEnd),
                            strand=hg38.rmsk$strand)
## To fetch ensembl gene exons from BioMart
library(biomaRt)
ensembl <- useMart(biomart="ENSEMBL_MART_ENSEMBL",
                   host="dec2015.archive.ensembl.org")
ensembl <-  useDataset("hsapiens_gene_ensembl",mart=ensembl)
attributes <- listAttributes(ensembl)
exons <- getBM(attributes=c("chromosome_name", "exon_chrom_start",
                            "exon_chrom_end", "strand"), 
               mart=ensembl)
exonsRanges <- GRanges(seqnames=exons$chromosome_name,
                       ranges=IRanges(start=exons$exon_chrom_start,
                                      end=exons$exon_chrom_end),
                       strand=ifelse(exons$strand==1L, "+", "-")
                       )
seqlevelsStyle(exonsRanges) <- "UCSC"
## Use the existing Bioconductor annotation package for hg38
library(TxDb.Hsapiens.UCSC.hg38.knownGene)
exonsRanges <- exons(TxDb.Hsapiens.UCSC.hg38.knownGene)
```

The regions to filter out can also be provided in a bed file.
To import the bed file into *GRanges* in `R`,
*rtracklayer* provides a general function
`import.bed` to do that.
Since only the chromosome names, start and end coordinates are used in *CNEr*,
we provide a more efficient `readBed` function.

```
## Existing bed file for chr6:24,000,000..27,000,000 of Zebrafish danRer10
bedDanRer10Fn <- file.path(system.file("extdata", package="CNEr"), 
                           "filter_regions.danRer10.bed")
danRer10Filter <- readBed(bedDanRer10Fn)
danRer10Filter
```

```
## GRanges object with 5996 ranges and 0 metadata columns:
##          seqnames            ranges strand
##             <Rle>         <IRanges>  <Rle>
##      [1]     chr6 24000008-24000253      +
##      [2]     chr6 24000254-24000332      +
##      [3]     chr6 24000499-24000550      +
##      [4]     chr6 24000554-24000670      +
##      [5]     chr6 24000671-24000722      +
##      ...      ...               ...    ...
##   [5992]     chr6 26998029-26998373      +
##   [5993]     chr6 26998414-26998431      +
##   [5994]     chr6 26998432-26998526      +
##   [5995]     chr6 26998687-26998833      +
##   [5996]     chr6 26999116-26999802      +
##   -------
##   seqinfo: 1 sequence from an unspecified genome; no seqlengths
```

```
## Existing bed file for alignment region in Human hg38 against
## chr6:24,000,000..27,000,000 of danRer10
bedHg38Fn <- file.path(system.file("extdata", package="CNEr"), 
                       "filter_regions.hg38.bed")
hg38Filter <- readBed(bedHg38Fn)
hg38Filter
```

```
## GRanges object with 413 ranges and 0 metadata columns:
##         seqnames              ranges strand
##            <Rle>           <IRanges>  <Rle>
##     [1]     chr1   26817474-26819341      +
##     [2]     chr1 156772018-156772158      +
##     [3]     chr1 156772160-156772358      +
##     [4]     chr1 156772495-156772637      +
##     [5]     chr1 156774135-156774472      +
##     ...      ...                 ...    ...
##   [409]     chr9 134045293-134045421      +
##   [410]     chr9 134048002-134048454      +
##   [411]     chr9 134051562-134051709      +
##   [412]     chr9 134052306-134052443      +
##   [413]     chr9 134053265-134053590      +
##   -------
##   seqinfo: 17 sequences from an unspecified genome; no seqlengths
```

## 3.3 Creating a `CNE` class

We designed a `CNE` class to store all metadata of running the pipeline for identifying a set of CNEs between two species, including the intermediate and final results.
`CNE` can be created by providing the paths of the twoBit files of two assemblies, and
the paths of axt files, with each assembly as reference.

```
## Here we have the twoBit files from Bioconductor package
## BSgenome.Drerio.UCSC.danRer10 and BSgenome.Hsapiens.UCSC.hg38
cneDanRer10Hg38 <- CNE(
  assembly1Fn=file.path(system.file("extdata",
                                    package="BSgenome.Drerio.UCSC.danRer10"),
                        "single_sequences.2bit"),
  assembly2Fn=file.path(system.file("extdata",
                                    package="BSgenome.Hsapiens.UCSC.hg38"),
                        "single_sequences.2bit"),
  axt12Fn=axtFilesDanRer10Hg38, axt21Fn=axtFilesHg38DanRer10,
  cutoffs1=8L, cutoffs2=4L)
cneDanRer10Hg38
```

```
## An object of class "CNE"
## Slot "assembly1Fn":
## [1] "/home/biocbuild/bbs-3.7-bioc/R/library/BSgenome.Drerio.UCSC.danRer10/extdata/single_sequences.2bit"
## 
## Slot "assembly2Fn":
## [1] "/home/biocbuild/bbs-3.7-bioc/R/library/BSgenome.Hsapiens.UCSC.hg38/extdata/single_sequences.2bit"
## 
## Slot "axt12Fn":
## [1] "/tmp/RtmpEcax9n/Rinst3607550d3f19/CNEr/extdata/danRer10.hg38.net.axt"
## 
## Slot "axt21Fn":
## [1] "/tmp/RtmpEcax9n/Rinst3607550d3f19/CNEr/extdata/hg38.danRer10.net.axt"
## 
## Slot "window":
## [1] 50
## 
## Slot "identity":
## [1] 50
## 
## Slot "CNE12":
## GRangePairs object with 0 pairs and 0 metadata columns:
##        first    second
##    <GRanges> <GRanges>
## 
## Slot "CNE21":
## GRangePairs object with 0 pairs and 0 metadata columns:
##        first    second
##    <GRanges> <GRanges>
## 
## Slot "CNEMerged":
## GRangePairs object with 0 pairs and 0 metadata columns:
##        first    second
##    <GRanges> <GRanges>
## 
## Slot "CNEFinal":
## GRangePairs object with 0 pairs and 0 metadata columns:
##        first    second
##    <GRanges> <GRanges>
## 
## Slot "aligner":
## [1] "blat"
## 
## Slot "cutoffs1":
## [1] 8
## 
## Slot "cutoffs2":
## [1] 4
```

**Note**: the order of assemblies when creating CNE object is important.
Here we have danRer10 as assembly1 and hg38 as assembly2.
Then the `axt12Fn` contains the axt alignment with assembly1 danRer10 as reference and `axt21Fn` contains the alignment with assembly2 hg38 as reference.
The `cutoffs1` and `cutoffs2` are the maximal number of hits during the realignment in later steps.
Because zebrafish has undergone additional whole genome duplication compared to human, the cutoffs of zebrafish also doubles the cutoffs of human.

# 4 CNE identification

In this section, we will go through the details of CNE identification.

## 4.1 Scan axt alignments

Detecting CNEs highly relies on the whole-genome pairwise net alignments.
To correct the bias of a chosen genome (which bias?) and
capture the duplicated CNEs during genome evolution,
we scan two sets of nets for each pairwise comparison,
one as reference from each of the genomes.

We identify CNEs by scanning the alignments for regions with
at least **I** identities over **C** alignment columns.
Because different genes and loci may favor various similarity scores,
we usually scan at two diffrent window sizes 30 and 50 with
several similarity criterias (**I/C**) range from 70% to 100%.

```
identities <- c(45L, 48L, 49L)
windows <- c(50L, 50L, 50L)
## Here danRer10Filter is tFilter since danRer10 is assembly1
cneListDanRer10Hg38 <- ceScan(x=cneDanRer10Hg38, tFilter=danRer10Filter,
                              qFilter=hg38Filter,
                              window=windows, identity=identities)
```

```
## The number of axt files 1
## The number of axt alignments is 352
## The number of axt files 1
## The number of axt alignments is 50
```

At this stage, a list of `CNE` is returned from `ceScan`, which contains the preliminary two sets of CNEs from a pair of axt alignments.
We can examine the intermediate CNEs by

```
## CNEs from the alignments with danRer10 as reference
CNE12(cneListDanRer10Hg38[["45_50"]])
```

```
## GRangePairs object with 74 pairs and 2 metadata columns:
##                           first                     second |     score
##                       <GRanges>                  <GRanges> | <numeric>
##    [1] chr6:26746284-26746334:+ chr3:137269941-137269991:+ |      90.2
##    [2] chr6:26745047-26745455:+ chr3:137264717-137265124:+ |     95.11
##    [3] chr6:26708061-26708129:+ chr3:137294941-137295008:- |      91.3
##    [4] chr6:26699009-26699078:+ chr3:137329801-137329870:- |     92.86
##    [5] chr6:26699080-26699274:+ chr3:137329608-137329799:- |     90.26
##    ...                      ...                        ... .       ...
##   [70] chr6:24605694-24605830:+   chr1:90841095-90841232:- |     92.75
##   [71] chr6:24605831-24605936:+   chr1:90840976-90841082:- |     90.65
##   [72] chr6:24606573-24606626:+   chr1:90840328-90840381:- |     90.74
##   [73] chr6:24599740-24599806:+   chr1:90856700-90856765:- |     92.54
##   [74] chr6:24580009-24580099:+   chr1:90944046-90944137:- |     90.22
##                cigar
##          <character>
##    [1]           51M
##    [2]    218M1I190M
##    [3]      47M1I21M
##    [4]           70M
##    [5] 82M1I96M2I14M
##    ...           ...
##   [70]      128M1D9M
##   [71]       99M1D7M
##   [72]           54M
##   [73]      33M1I33M
##   [74]      12M1D79M
```

```
## CNEs from the alignments with hg38 as reference
CNE21(cneListDanRer10Hg38[["45_50"]])
```

```
## GRangePairs object with 4 pairs and 2 metadata columns:
##                            first                   second |     score
##                        <GRanges>                <GRanges> | <numeric>
##   [1] chr3:137523038-137523102:+ chr6:26638744-26638808:+ |     87.69
##   [2] chr3:137523122-137523187:+ chr6:26638826-26638891:+ |     89.39
##   [3] chr3:137269941-137269991:+ chr6:26746284-26746334:+ |      90.2
##   [4] chr3:137264717-137265124:+ chr6:26745047-26745455:+ |     95.11
##             cigar
##       <character>
##   [1]         65M
##   [2]         66M
##   [3]         51M
##   [4]  218M1D190M
```

In the result table, even though the strand for query element can be negative,
the coordinate for that query element is already on the positive strand.

It is essential to scan two sets of pairwise net alignments with each assembly as reference, in order not to miss any duplicated elements in either lineage.
This is particularly important for the comparison between teleost fishes and other vertebrates, because one (for instance, the case of zebrafish) or two (the case of common carp) extra whole genome duplications occured.

## 4.2 Merge CNEs

As we perform two rounds of CNE detection with each genome as reference,
some conserved elements overlap on both genomes and should be removed.
Elements, however, that overlap only on one of the genomes are kept,
so that duplicated elements remain distinct.

```
cneMergedListDanRer10Hg38 <- lapply(cneListDanRer10Hg38, cneMerge)
```

## 4.3 Realignment of CNEs

Some CNEs might be unannotated repeats.
To remove them, currently we use **blat** (W James Kent 2002) to realign
each sequence of CNEs against the respective genomes.
When the number of matches exceeds a certain threshold, for instance 8,
that CNE will be discarded.

This step can be very time-consuming when the number of CNEs is large.
Other alignment methods could also be considered, for example Bowtie2 or BWA (provided that they are installed on the user’s machine)

```
cneFinalListDanRer10Hg38 <- lapply(cneMergedListDanRer10Hg38, blatCNE)
```

## 4.4 CNE storage and query

As the computation of CNEs from the whole pipeline and
the preparation of annotation package can be very time-consuming,
for a smoother visualisation experience,
we decided to use a local SQLite database in order to store CNEs.

Since the CNEs `data.frame` is a table, it can be imported into a SQL table.
To speed up the query from the SQL database,
the bin indexing system is adopted.
For more information, please refer to the paper (W J Kent et al. 2002)
and genomewiki.

```
## on individual tables
dbName <- tempfile()
data(cneFinalListDanRer10Hg38)
tableNames <- paste("danRer10", "hg38", names(cneFinalListDanRer10Hg38),
                    sep="_")
for(i in 1:length(cneFinalListDanRer10Hg38)){
    saveCNEToSQLite(cneFinalListDanRer10Hg38[[i]], dbName, tableNames[i],
                    overwrite=TRUE)
}
```

When querying results from the local SQLite database based on the chr,
coordinates and other criterias,
a `GRanges` object is returned.

```
chr <- "chr6"
start <- 24000000L
end <-  27000000L
minLength <- 50L
tableName <- "danRer10_hg38_45_50"
fetchedCNERanges <- readCNERangesFromSQLite(dbName, tableName, chr, 
                                            start, end, whichAssembly="first",
                                            minLength=minLength)
fetchedCNERanges
```

```
## GRangePairs object with 70 pairs and 0 metadata columns:
##                         first                   second
##                     <GRanges>                <GRanges>
##    [1] chr6:26708061-26708129 chr3:137294941-137295008
##    [2] chr6:26699009-26699078 chr3:137329801-137329870
##    [3] chr6:26699080-26699274 chr3:137329608-137329799
##    [4] chr6:26688342-26688673 chr3:137406806-137407138
##    [5] chr6:26677966-26678081 chr3:137467520-137467634
##    ...                    ...                      ...
##   [66] chr6:24580009-24580099   chr1:90944046-90944137
##   [67] chr6:26638744-26638808 chr3:137523038-137523102
##   [68] chr6:26638826-26638891 chr3:137523122-137523187
##   [69] chr6:26746284-26746334 chr3:137269941-137269991
##   [70] chr6:26745047-26745455 chr3:137264717-137265124
```

## 4.5 CNE length distribution

As the lengths of CNEs (Salerno, Havlak, and Miller 2006) and the distances between consecutive elements (Polychronopoulos, Sellis, and Almirantis 2014) exhibit power-law distributions, we implemented a function that might be useful in showing interesting patterns in the distribution of the identified elements.

```
dbName <- file.path(system.file("extdata", package="CNEr"),
                    "danRer10CNE.sqlite")
tAssemblyFn <- file.path(system.file("extdata",
                         package="BSgenome.Drerio.UCSC.danRer10"),
                         "single_sequences.2bit")
qAssemblyFn <- file.path(system.file("extdata",
                         package="BSgenome.Hsapiens.UCSC.hg38"),
                         "single_sequences.2bit")
cneGRangePairs <- readCNERangesFromSQLite(dbName=dbName, 
                                          tableName="danRer10_hg38_45_50",
                                          tAssemblyFn=tAssemblyFn,
                                          qAssemblyFn=qAssemblyFn)
plotCNEWidth(cneGRangePairs)
```

## 4.6 Genomic distribution of CNEs along the chromosome

CNEs tend to form clusters. A quick check of the genomic distribution of CNEs
is available.

```
plotCNEDistribution(first(cneGRangePairs))
```

## 4.7 Output of bed and bedGraph files

For visualisation in other Genome Browser, we provide functions to generate the CNE in bed files and CNE density in bedGraph files.
For example, to get the first 1000 coordinates of CNEs:

```
makeCNEDensity(cneGRangePairs[1:1000])
```

# 5 CNEs visualisation

To visualise CNEs alongside other gene annotations,
we choose to use the Bioconductor package *Gviz* in this vignette.
*Gviz*, based on the *grid* graphics scheme,
is a very powerful package for plotting data and annotation information
along genomic coordinates.
The functionality of integrating publicly available genome annotation data,
such as UCSC or Ensembl,
significantly reduced the burden of preparing annotations for common assemblies.
Since the Bioconductor release 2.13 of *Gviz*,
it provides the data track in horizon plot,
which exactly meets our needs for visualisation of CNEs density plots.
For more detailed usage, please check the vignette or manual of *Gviz*.

Another option for visualisation is the package *ggbio*,
which is based on *ggplot2*.
The advantage of *ggbio* is the simplicity of
adding any customised *ggplot2* style track
into the plot without tuning the coordinate systems.
The densities generated in the following section can be easily plot in the
horizon plot.
A short straightforward tutorial regarding horizon plot
in `ggplot2` format
is available from
http://timelyportfolio.blogspot.co.uk/2012/08/horizon-on-ggplot2.html.

## 5.1 Gene annotation visualisation

For the example case of hg38 vs danRer10 in this vignette,
we choose danRer10 as the reference and
show the range of developmental gene **barhl2** and **sox14**.

```
library(Gviz)
library(biomaRt)
genome <- "danRer10"
axisTrack <- GenomeAxisTrack()
cpgIslands <- UcscTrack(genome=genome, chromosome=chr,
                        track="cpgIslandExt", from=start, to=end,
                        trackType="AnnotationTrack", start="chromStart",
                        end="chromEnd", id="name", shape="box",
                        showId=FALSE,
                        fill="#006400", name="CpG",
                        background.title="brown")
refGenes <- UcscTrack(genome=genome, chromosome=chr,
                      track="refGene", from=start, to=end,
                      trackType="GeneRegionTrack", rstarts="exonStarts",
                      rends="exonEnds", gene="name2", symbol="name2",
                      transcript="name", strand="strand", fill="#8282d2",
                      name="refSeq Genes", collapseTranscripts=TRUE,
                      showId=TRUE, background.title="brown")
ensembl <- useMart(biomart="ENSEMBL_MART_ENSEMBL",
                   host="dec2015.archive.ensembl.org")
ensembl <-  useDataset("drerio_gene_ensembl",mart=ensembl)
biomTrack <- BiomartGeneRegionTrack(genome=genome, chromosome=chr, 
                                    biomart=ensembl,
                                    start=start , end=end, name="Ensembl Genes")
```

```
library(Gviz)
plotTracks(list(axisTrack, cpgIslands, refGenes), 
           collapseTranscripts=TRUE, shape="arrow",
           transcriptAnnotation="symbol")
```

It is also possible to plot the annotation from an ordinary `R` object,
such as `data.frame`, `GRanges`, `IRanges`
or even from a local file.
Usually the **gff** file containing the gene annotation can be processed by
*Gviz* directly.
For more details, please look into the vignette of *Gviz*.

## 5.2 CNEs horizon plot

```
dbName <- file.path(system.file("extdata", package="CNEr"),
                    "danRer10CNE.sqlite")
genome <- "danRer10"
windowSize <- 200L
minLength <- 50L
cneDanRer10Hg38_21_30 <- 
  CNEDensity(dbName=dbName, 
             tableName="danRer10_hg38_21_30",
             whichAssembly="first", chr=chr, start=start,
             end=end, windowSize=windowSize, 
             minLength=minLength)
cneDanRer10Hg38_45_50 <-
  CNEDensity(dbName=dbName, 
             tableName="danRer10_hg38_45_50", 
             whichAssembly="first", chr=chr, start=start,
             end=end, windowSize=windowSize, 
             minLength=minLength)
cneDanRer10Hg38_49_50 <-
  CNEDensity(dbName=dbName, 
             tableName="danRer10_hg38_49_50", 
             whichAssembly="first", chr=chr, start=start,
             end=end, windowSize=windowSize, 
             minLength=minLength)
cneDanRer10AstMex102_48_50 <-
  CNEDensity(dbName=dbName, 
             tableName="AstMex102_danRer10_48_50",
             whichAssembly="second", chr=chr, start=start,
             end=end, windowSize=windowSize, 
             minLength=minLength)
cneDanRer10CteIde1_75_75 <-
  CNEDensity(dbName=dbName, 
             tableName="cteIde1_danRer10_75_75", 
             whichAssembly="second", chr=chr, start=start,
             end=end, windowSize=windowSize, 
             minLength=minLength)
```

```
dTrack1 <- DataTrack(range=cneDanRer10Hg38_21_30,
                     genome=genome, type="horiz", 
                     horizon.scale=max(cneDanRer10Hg38_21_30$score)/3, 
                     fill.horizon=c("#B41414", "#E03231", "#F7A99C", 
                                    "yellow", "orange", "red"), 
                     name="human 21/30", background.title="brown")
dTrack2 <- DataTrack(range=cneDanRer10Hg38_45_50,
                     genome=genome, type="horiz", 
                     horizon.scale=max(cneDanRer10Hg38_45_50$score)/2, 
                     fill.horizon=c("#B41414", "#E03231", "#F7A99C", 
                                    "yellow", "orange", "red"), 
                     name="human 45/50", background.title="brown")
dTrack3 <- DataTrack(range=cneDanRer10Hg38_49_50,
                     genome=genome, type="horiz", 
                     horizon.scale=max(cneDanRer10Hg38_21_30$score)/3, 
                     fill.horizon=c("#B41414", "#E03231", "#F7A99C", 
                                    "yellow", "orange", "red"), 
                     name="human 49/50", background.title="brown")
dTrack4 <- DataTrack(range=cneDanRer10AstMex102_48_50,
                     genome=genome, type="horiz", 
                     horizon.scale=max(cneDanRer10Hg38_21_30$score)/3, 
                     fill.horizon=c("#B41414", "#E03231", "#F7A99C", 
                                    "yellow", "orange", "red"), 
                     name="blind cave fish 48/50", background.title="brown")
dTrack5 <- DataTrack(range=cneDanRer10CteIde1_75_75,
                     genome=genome, type="horiz", 
                     horizon.scale=max(cneDanRer10CteIde1_75_75$score)/3, 
                     fill.horizon=c("#B41414", "#E03231", "#F7A99C", 
                                    "yellow", "orange", "red"), 
                     name="grass carp 75/75", background.title="brown")
```

```
ht <- HighlightTrack(trackList=list(refGenes, dTrack5, dTrack4, 
                                    dTrack1, dTrack2, dTrack3), 
                     start=c(24200000, 25200000, 26200000), 
                     end=c(25100000, 26150000, 27000000),
                     chromosome =chr)
plotTracks(list(axisTrack, cpgIslands, ht),
           collapseTranscripts=TRUE, shape="arrow",
           transcriptAnnotation="symbol",
           from=24000000, to=27000000)
```

From this horizon plot and when comparing Zebrafish with Human as a reference genome,
we notice that the genes barhl2, lmo4b and sox14 are surrounded by the density peaks of CNEs.

# 6 Conclusions

*CNEr* efficiently identifies CNEs
and handles the corresponding objects conveniently in R.  
Horizon plot shows a superior dynamic range to the standard density plots,
simultaneously revealing CNE clusters characterized
by vastly different levels of sequence conservation.
Such CNE density plots generated using precise locations of CNEs
can be used to identify genes involved in developmental regulation,
even for novel genes that are not yet annotated.

The following is the session info that generated this vignette:

```
  sessionInfo()
```

```
## R version 3.5.0 (2018-04-23)
## Platform: x86_64-pc-linux-gnu (64-bit)
## Running under: Ubuntu 16.04.4 LTS
## 
## Matrix products: default
## BLAS: /home/biocbuild/bbs-3.7-bioc/R/lib/libRblas.so
## LAPACK: /home/biocbuild/bbs-3.7-bioc/R/lib/libRlapack.so
## 
## locale:
##  [1] LC_CTYPE=en_US.UTF-8       LC_NUMERIC=C              
##  [3] LC_TIME=en_US.UTF-8        LC_COLLATE=C              
##  [5] LC_MONETARY=en_US.UTF-8    LC_MESSAGES=en_US.UTF-8   
##  [7] LC_PAPER=en_US.UTF-8       LC_NAME=C                 
##  [9] LC_ADDRESS=C               LC_TELEPHONE=C            
## [11] LC_MEASUREMENT=en_US.UTF-8 LC_IDENTIFICATION=C       
## 
## attached base packages:
##  [1] grid      stats4    parallel  stats     graphics  grDevices utils    
##  [8] datasets  methods   base     
## 
## other attached packages:
##  [1] Gviz_1.24.0                         BSgenome.Ggallus.UCSC.galGal3_1.4.0
##  [3] BSgenome.Hsapiens.UCSC.hg19_1.4.0   BSgenome_1.48.0                    
##  [5] rtracklayer_1.40.2                  Biostrings_2.48.0                  
##  [7] XVector_0.20.0                      GenomicRanges_1.32.3               
##  [9] GenomeInfoDb_1.16.0                 IRanges_2.14.10                    
## [11] S4Vectors_0.18.2                    BiocGenerics_0.26.0                
## [13] CNEr_1.16.1                         BiocStyle_2.8.2                    
## 
## loaded via a namespace (and not attached):
##  [1] ProtGenerics_1.12.0         bitops_1.0-6               
##  [3] matrixStats_0.53.1          bit64_0.9-7                
##  [5] RColorBrewer_1.1-2          progress_1.1.2             
##  [7] httr_1.3.1                  rprojroot_1.3-2            
##  [9] tools_3.5.0                 backports_1.1.2            
## [11] R6_2.2.2                    rpart_4.1-13               
## [13] Hmisc_4.1-1                 DBI_1.0.0                  
## [15] lazyeval_0.2.1              colorspace_1.3-2           
## [17] nnet_7.3-12                 gridExtra_2.3              
## [19] prettyunits_1.0.2           curl_3.2                   
## [21] bit_1.1-14                  compiler_3.5.0             
## [23] Biobase_2.40.0              htmlTable_1.12             
## [25] DelayedArray_0.6.0          labeling_0.3               
## [27] bookdown_0.7                checkmate_1.8.5            
## [29] scales_0.5.0                readr_1.1.1                
## [31] stringr_1.3.1               digest_0.6.15              
## [33] Rsamtools_1.32.0            foreign_0.8-70             
## [35] rmarkdown_1.9               R.utils_2.6.0              
## [37] dichromat_2.0-0             base64enc_0.1-3            
## [39] pkgconfig_2.0.1             htmltools_0.3.6            
## [41] ensembldb_2.4.1             htmlwidgets_1.2            
## [43] rlang_0.2.1                 rstudioapi_0.7             
## [45] RSQLite_2.1.1               VGAM_1.0-5                 
## [47] BiocParallel_1.14.1         acepack_1.4.1              
## [49] R.oo_1.22.0                 VariantAnnotation_1.26.0   
## [51] RCurl_1.95-4.10             magrittr_1.5               
## [53] GO.db_3.6.0                 GenomeInfoDbData_1.1.0     
## [55] Formula_1.2-3               Matrix_1.2-14              
## [57] Rcpp_0.12.17                munsell_0.4.3              
## [59] R.methodsS3_1.7.1           stringi_1.2.2              
## [61] yaml_2.1.19                 SummarizedExperiment_1.10.1
## [63] zlibbioc_1.26.0             plyr_1.8.4                 
## [65] blob_1.1.1                  lattice_0.20-35            
## [67] splines_3.5.0               GenomicFeatures_1.32.0     
## [69] annotate_1.58.0             hms_0.4.2                  
## [71] KEGGREST_1.20.0             knitr_1.20                 
## [73] pillar_1.2.3                reshape2_1.4.3             
## [75] biomaRt_2.36.1              XML_3.98-1.11              
## [77] evaluate_0.10.1             biovizBase_1.28.0          
## [79] latticeExtra_0.6-28         data.table_1.11.4          
## [81] png_0.1-7                   gtable_0.2.0               
## [83] poweRlaw_0.70.1             assertthat_0.2.0           
## [85] ggplot2_2.2.1               xfun_0.1                   
## [87] xtable_1.8-2                AnnotationFilter_1.4.0     
## [89] survival_2.42-3             tibble_1.4.2               
## [91] GenomicAlignments_1.16.0    AnnotationDbi_1.42.1       
## [93] memoise_1.1.0               cluster_2.0.7-1
```

# References

Kent, W James. 2002. “BLAT–the BLAST-like alignment tool.” *Genome Research* 12 (4):656–64.

Kent, W J, C W Sugnet, T S Furey, K M Roskin, T H Pringle, A M Zahler, and a D Haussler. 2002. “The Human Genome Browser at UCSC.” *Genome Research* 12 (6):996–1006.

Polychronopoulos, Dimitris, Diamantis Sellis, and Yannis Almirantis. 2014. “Conserved Noncoding Elements Follow Power-Law-Like Distributions in Several Genomes as a Result of Genome Dynamics.” *PloS One* 9 (5). Public Library of Science:e95437.

Salerno, William, Paul Havlak, and Jonathan Miller. 2006. “Scale-Invariant Structure of Strongly Conserved Sequence in Genomic Intersections and Alignments.” *Proceedings of the National Academy of Sciences* 103 (35). National Acad Sciences:13121–5.

Sandelin, Albin, Peter Bailey, Sara Bruce, Pär G Engström, Joanna M Klos, Wyeth W Wasserman, Johan Ericson, and Boris Lenhard. 2004. “Arrays of ultraconserved non-coding regions span the loci of key developmental genes in vertebrate genomes.” *BMC Genomics* 5 (1):99.

Woolfe, Adam, Martin Goodson, Debbie K Goode, Phil Snell, Gayle K McEwen, Tanya Vavouri, Sarah F Smith, Phil North, Heather Callaway, and Krys Kelly. 2004. “Highly conserved non-coding sequences are associated with vertebrate development.” *PLoS Biology* 3 (1):e7.
