# Supplementary material for: CNEr: A toolkit for exploring extreme noncoding conservation
Source: PLoS Comput Biol. 2019 Aug 26;15(8):e1006940. doi: 10.1371/journal.pcbi.1006940 (PMC6730951; doi:10.1371/journal.pcbi.1006940)
Supplement: S1 Code — (ZIP) [file pcbi.1006940.s013.zip › S1Code/package/inst/doc/PairwiseWholeGenomeAlignment.html]

Pairwise whole genome alignment


# Pairwise whole genome alignment

Ge Tan

#### *31 May 2018*

#### Abstract

Pipeline of generating pairwise whole genome alignment.

#### Package

CNEr 1.16.1

# Contents

- 1 Introduction
- 2 Prerequisite
- 3 Aligning
  - 3.1 LASTZ aligner
  - 3.2 last aligner
  - 3.3 YASS aligner
- 4 Chaining:
- 5 Netting:
- 6 axtNet
- 7 Session info

# 1 Introduction

*CNEr* identifies conserved noncoding elments (CNEs) from pairwise whole genome alignment (*net.axt* files) of two species.
UCSC has provided alignments between many species on the downloads,
hence it is highly recommended to use their alignments when available.
When the alignments of some new assemblies/species are not availble from UCSC yet, this vignette describes the pipeline of generating the alignments merely from soft-masked *2bit* files or *fasta* files.
This vignette is based on the genomewiki from UCSC.

**Note:**

- The whole pipeline relies on Kent’s utilities.
  Since Kent’s utilities don’t support Windows platform, this pipeline also only works on Linux and Unix platform.
- Many of the functions in this pipeline are just wrapper functions, in order to simplfy the settings and work consistently with the rest of CNEs pipeline within *R* environment.
- Due to large file size of the genome assemblies, the commands in this vignette don’t run during the vignette compilation. Users have to set the correct paths on their machine in order to make these commands work.

# 2 Prerequisite

List of external softwares have to be installed on the machine:
\* The sequence alignment program LASTZ
\* Kent Utilities. In this pipeline, *lavToPsl*, *axtChain*, *chainMergeSort*, *chainPreNet*, *chainNet*, *netSyntenic*, *netToAxt*, *axtSort* are essential. netClass is optional.

# 3 Aligning

Here, as an example, we will get the pairwise alignment only on “chr1”, “chr2” and “chr3” between zebrafish(*danRer10*) and human(*hg38*).

## 3.1 LASTZ aligner

First we need to download the *2bit* files from UCSC, and set the appropriate paths of `assemblyTarget`, `assemblyQuery` and intermediate files.
Then we can run `lastz` function to generate the *lav* files.

```
## lastz aligner
assemblyDir <- "/Users/gtan/OneDrive/Project/CSC/CNEr/2bit"
axtDir <- "/Users/gtan/OneDrive/Project/CSC/CNEr/axt"
assemblyTarget <- file.path(system.file("extdata",
                            package="BSgenome.Drerio.UCSC.danRer10"),
                            "single_sequences.2bit")
assemblyQuery <- file.path(system.file("extdata",
                                       package="BSgenome.Hsapiens.UCSC.hg38"),
                           "single_sequences.2bit")
lavs <- lastz(assemblyTarget, assemblyQuery, 
              outputDir=axtDir,
              chrsTarget=c("chr1", "chr2", "chr3"),
              chrsQuery=c("chr1", "chr2", "chr3"),
              distance="far", mc.cores=4)

## lav files to psl files conversion
psls <- lavToPsl(lavs, removeLav=FALSE, binary="lavToPsl")
```

One essential argument here is the `distance`. It determines the scoring matrix to use in `lastz` aligner. See `?scoringMatrix` for more details.

**Note:**
This step may encounter difficulties if the two assemblies are too fragmented, because there can be millions of combinations of the chromosomes/scaffolds. The `lastz` is overwhelmed with reading small pieces from assembly for each combination, rather than doing actual alignment.
In this case, another aligner last is recommended and introduced in nex section.

## 3.2 last aligner

`last` aligner is considered faster and memory efficient.
It creates *maf* file, which can converted to *psl* files.
Then the same following processes can be used on *psl* files.

Different from `lastz`, `last` aligner starts with *fasta* files.
The target genome sequence has to build the *index* file first, and then align with the query genome sequence.

```
## Build the lastdb index
system2(command="lastdb", args=c("-c", file.path(assemblyDir, "danRer10"),
                                 file.path(assemblyDir, "danRer10.fa")))

## Run last aligner
lastal(db=file.path(assemblyDir, "danRer10"),
       queryFn=file.path(assemblyDir, "hg38.fa"),
       outputFn=file.path(axtDir, "danRer10.hg38.maf"),
       distance="far", binary="lastal", mc.cores=4L)

## maf to psl 
psls <- file.path(axtDir, "danRer10.hg38.psl")
system2(command="maf-convert", args=c("psl", 
                                      file.path(axtDir, "danRer10.hg38.maf"),
                                      ">", psls))
```

## 3.3 YASS aligner

Another alternative of alignment software is
YASS.
It may be added into this pipeline after we test the performance.

# 4 Chaining:

If two matching alignments next to each other are close enough, they are joined into one fragment.
Then these *chain* files are sorted and combined into one big file.

```
## Join close alignments
chains <- axtChain(psls, assemblyTarget=assemblyTarget,
                   assemblyQuery=assemblyQuery, distance="far",
                   removePsl=FALSE, binary="axtChain")

## Sort and combine
allChain <- chainMergeSort(chains, assemblyTarget, assemblyQuery,
              allChain=file.path(axtDir,
                         paste0(sub("\\.2bit$", "", basename(assemblyTarget),
                                    ignore.case=TRUE), ".", 
                                sub("\\.2bit$", "", basename(assemblyQuery), 
                                    ignore.case=TRUE), ".all.chain")),
                           removeChains=FALSE, binary="chainMergeSort")
```

# 5 Netting:

In this step, first we filter out chains that are unlikely to be netted by `chainPreNet`.
During the alignment, every genomic fragment can match with several others, and certainly we want to keep the best one.
This is done by `chainNet`.
Then we add the synteny information with `netSyntenic`.

```
## Filtering out chains
allPreChain <- chainPreNet(allChain, assemblyTarget, assemblyQuery,
                           allPreChain=file.path(axtDir,
                                      paste0(sub("\\.2bit$", "", 
                                                 basename(assemblyTarget),
                                                 ignore.case = TRUE), ".", 
                                             sub("\\.2bit$", "",
                                                 basename(assemblyQuery),
                                                 ignore.case = TRUE),
                                                 ".all.pre.chain")),
                           removeAllChain=FALSE, binary="chainPreNet")

## Keep the best chain and add synteny information
netSyntenicFile <- chainNetSyntenic(allPreChain, assemblyTarget, assemblyQuery,
                     netSyntenicFile=file.path(axtDir,
                                               paste0(sub("\\.2bit$", "",
                                                      basename(assemblyTarget),
                                                      ignore.case = TRUE), ".",
                                                      sub("\\.2bit$", "",
                                                      basename(assemblyQuery),
                                                      ignore.case = TRUE),
                                               ".noClass.net")),
                     binaryChainNet="chainNet", binaryNetSyntenic="netSyntenic")
```

# 6 axtNet

As the last step, we create the *.net.axt* file from the previous *net* and *chain* files.

```
netToAxt(netSyntenicFile, allPreChain, assemblyTarget, assemblyQuery,
         axtFile=file.path(axtDir,
                           paste0(sub("\\.2bit$", "",
                                      basename(assemblyTarget),
                                      ignore.case = TRUE), ".",
                                  sub("\\.2bit$", "",
                                      basename(assemblyQuery),
                                      ignore.case = TRUE),
                                  ".net.axt")),
             removeFiles=FALSE,
             binaryNetToAxt="netToAxt", binaryAxtSort="axtSort")
```

# 7 Session info

Here is the output of `sessionInfo()` on the system on which this
document was compiled:

```
## R version 3.5.0 (2018-04-23)
## Platform: x86_64-pc-linux-gnu (64-bit)
## Running under: Ubuntu 16.04.4 LTS
## 
## Matrix products: default
## BLAS: /home/biocbuild/bbs-3.7-bioc/R/lib/libRblas.so
## LAPACK: /home/biocbuild/bbs-3.7-bioc/R/lib/libRlapack.so
## 
## locale:
##  [1] LC_CTYPE=en_US.UTF-8       LC_NUMERIC=C              
##  [3] LC_TIME=en_US.UTF-8        LC_COLLATE=C              
##  [5] LC_MONETARY=en_US.UTF-8    LC_MESSAGES=en_US.UTF-8   
##  [7] LC_PAPER=en_US.UTF-8       LC_NAME=C                 
##  [9] LC_ADDRESS=C               LC_TELEPHONE=C            
## [11] LC_MEASUREMENT=en_US.UTF-8 LC_IDENTIFICATION=C       
## 
## attached base packages:
##  [1] grid      stats4    parallel  stats     graphics  grDevices utils    
##  [8] datasets  methods   base     
## 
## other attached packages:
##  [1] Gviz_1.24.0                         BSgenome.Ggallus.UCSC.galGal3_1.4.0
##  [3] BSgenome.Hsapiens.UCSC.hg19_1.4.0   BSgenome_1.48.0                    
##  [5] rtracklayer_1.40.2                  Biostrings_2.48.0                  
##  [7] XVector_0.20.0                      GenomicRanges_1.32.3               
##  [9] GenomeInfoDb_1.16.0                 IRanges_2.14.10                    
## [11] S4Vectors_0.18.2                    BiocGenerics_0.26.0                
## [13] CNEr_1.16.1                         BiocStyle_2.8.2                    
## 
## loaded via a namespace (and not attached):
##  [1] ProtGenerics_1.12.0         bitops_1.0-6               
##  [3] matrixStats_0.53.1          bit64_0.9-7                
##  [5] RColorBrewer_1.1-2          progress_1.1.2             
##  [7] httr_1.3.1                  rprojroot_1.3-2            
##  [9] tools_3.5.0                 backports_1.1.2            
## [11] R6_2.2.2                    rpart_4.1-13               
## [13] Hmisc_4.1-1                 DBI_1.0.0                  
## [15] lazyeval_0.2.1              colorspace_1.3-2           
## [17] nnet_7.3-12                 gridExtra_2.3              
## [19] prettyunits_1.0.2           curl_3.2                   
## [21] bit_1.1-14                  compiler_3.5.0             
## [23] Biobase_2.40.0              htmlTable_1.12             
## [25] DelayedArray_0.6.0          labeling_0.3               
## [27] bookdown_0.7                checkmate_1.8.5            
## [29] scales_0.5.0                readr_1.1.1                
## [31] stringr_1.3.1               digest_0.6.15              
## [33] Rsamtools_1.32.0            foreign_0.8-70             
## [35] rmarkdown_1.9               R.utils_2.6.0              
## [37] dichromat_2.0-0             base64enc_0.1-3            
## [39] pkgconfig_2.0.1             htmltools_0.3.6            
## [41] ensembldb_2.4.1             htmlwidgets_1.2            
## [43] rlang_0.2.1                 rstudioapi_0.7             
## [45] RSQLite_2.1.1               VGAM_1.0-5                 
## [47] BiocParallel_1.14.1         acepack_1.4.1              
## [49] R.oo_1.22.0                 VariantAnnotation_1.26.0   
## [51] RCurl_1.95-4.10             magrittr_1.5               
## [53] GO.db_3.6.0                 GenomeInfoDbData_1.1.0     
## [55] Formula_1.2-3               Matrix_1.2-14              
## [57] Rcpp_0.12.17                munsell_0.4.3              
## [59] R.methodsS3_1.7.1           stringi_1.2.2              
## [61] yaml_2.1.19                 SummarizedExperiment_1.10.1
## [63] zlibbioc_1.26.0             plyr_1.8.4                 
## [65] blob_1.1.1                  lattice_0.20-35            
## [67] splines_3.5.0               GenomicFeatures_1.32.0     
## [69] annotate_1.58.0             hms_0.4.2                  
## [71] KEGGREST_1.20.0             knitr_1.20                 
## [73] pillar_1.2.3                reshape2_1.4.3             
## [75] biomaRt_2.36.1              XML_3.98-1.11              
## [77] evaluate_0.10.1             biovizBase_1.28.0          
## [79] latticeExtra_0.6-28         data.table_1.11.4          
## [81] png_0.1-7                   gtable_0.2.0               
## [83] poweRlaw_0.70.1             assertthat_0.2.0           
## [85] ggplot2_2.2.1               xfun_0.1                   
## [87] xtable_1.8-2                AnnotationFilter_1.4.0     
## [89] survival_2.42-3             tibble_1.4.2               
## [91] GenomicAlignments_1.16.0    AnnotationDbi_1.42.1       
## [93] memoise_1.1.0               cluster_2.0.7-1
```
